# Avoidable mortality, clinical outcomes and determinants of premature death among adults with severe or profound intellectual disability: A population-based analysis of mortality data in England

**DOI:** 10.1371/journal.pmed.1005029

**Published:** 2026-08-17

**Authors:** Michael Kwan Leung Yu, Rory Sheehan, Nicholas Magill, Adam White, Lili Bokor, Jonathon Ding, Irene Tuffrey-Wijne, Umesh Chauhan, André Strydom

**Affiliations:** 1 Department of Forensic and Neurodevelopmental Sciences, Institute of Psychiatry, Psychology and Neuroscience, King’s College London, London, United Kingdom; 2 Oxleas NHS Foundation Trust, London, United Kingdom; 3 Department of Medical Statistics, Faculty of Epidemiology and Population Health, London School of Hygiene & Tropical Medicine, London, United Kingdom; 4 Department of Public Health, Children’s, Learning Disability and Mental Health Nursing, School of Nursing, Allied and Public Health, Kingston University, London, United Kingdom; 5 School of Medicine and Dentistry, University of Lancashire, Preston, United Kingdom; 6 South London and Maudsley NHS Foundation Trust, London, United Kingdom; University of Cambridge, UNITED KINGDOM OF GREAT BRITAIN AND NORTHERN IRELAND

## Abstract

**Background:**

Adults with intellectual disabilities experience significantly worse health outcomes and die younger than people without intellectual disability. People with severe or profound intellectual disability have particularly complex health needs. However, no large-scale population-based studies have specifically examined mortality patterns and factors associated with age at death in this group.

**Methods and findings:**

This population-based study used data from the English Learning from Lives and Deaths (LeDeR) mortality review programme and the Office for National Statistics (ONS) between 2021 and 2023. We included 1,301 adults with severe or profound intellectual disability, 2,626 adults with mild or moderate intellectual disability, and 536,311 adults from the general population. We collected sociodemographic, clinical, and mortality data. Key outcomes included co-occurring conditions, age at death, causes of death, avoidable mortality, and years of life lost. We used Cox regression and multiple linear regression to examine factors associated with age at death. We defined avoidable deaths as deaths before the age of 75 years due to preventable or treatable causes and adjudicated them by mapping the underlying cause of death to the Organisation for Economic Co-operation and Development (OECD) list of avoidable causes of death. Adults with severe or profound intellectual disability had a significantly higher prevalence of dysphagia (63.0%), epilepsy (36.1%) and visual problems (35.9%) compared with those with mild or moderate intellectual disability (*p* < 0.01 for each). The median age at death was markedly lower in this group (57.9 years) compared to those with mild or moderate intellectual disability (65.0 years) and the general population (81.9 years) (*p* < 0.001). We classified two-fifths of deaths among adults with severe or profound intellectual disability as avoidable (39.5%), resulting in 15,059.4 years of life lost. Severe or profound intellectual disability was associated with a younger age at death (adjusted mean difference −7.17 years; 95% confidence interval [CI] [−8.16, −6.18]; *p* < 0.001). A key limitation is that the cohort was restricted to mortality data, meaning our findings reflect factors associated with age at death rather than mortality risk.

**Conclusions:**

Our findings highlight the urgent need for tailored public health strategies, early interventions, and integrated care models to improve clinical outcomes for individuals with severe and profound intellectual disability.

## Author summary

**Why was this study done?:**

- People with intellectual disability die around 20 years younger than the general population, and many of these deaths are from conditions that could have been prevented or treated.
- People with severe or profound intellectual disability are likely to be most at risk, but no large-scale study had specifically examined their causes of death or avoidable mortality in this group.
- We used a population-based dataset in England that records deaths of people with intellectual disability, enabling us to compare outcomes by level of disability.

**What did the researchers do and find?:**

- We included 1,301 adults with severe or profound intellectual disability who died in England between 2021 and 2023 and compared them with 2,626 adults with mild or moderate intellectual disability, and 536,311 adults from the general population, who died over the same period.
- Adults with severe or profound intellectual disability died at a median age of 57.9 years, younger than those with mild or moderate intellectual disability (who died at a median of 65.0 years) and general population (81.9 years)
- Two in five deaths of adults with severe or profound intellectual disability were from avoidable causes, resulting in over 15,000 years of life lost.

**What do these findings mean?:**

- Two-thirds of avoidable deaths in people with severe or profound intellectual disability are from treatable conditions, particularly pneumonia and epilepsy, suggesting that better access to timely healthcare could save lives.
- Health services need to ensure that key interventions, including annual health checks, vaccination programmes, and timely management of conditions such as pneumonia and epilepsy, actively reach adults with severe or profound intellectual disability, with particular attention to those from ethnic minority backgrounds who face compounding disadvantages.
- The main limitation of our study is that we only included people who had already died, so our findings can tell us which groups died at younger ages, but they cannot tell us who is most likely to die early.

## Introduction

Intellectual disability, also known as learning disability in the United Kingdom (UK), arises during the developmental period and is characterised by significant limitations in cognitive ability, adaptive behaviour, and daily living skills [1,2]. Intellectual disability is present in approximately 1%–3% of the population [3], and it is estimated that around 1.3 million individuals in England have intellectual disability, including 977,000 adults [4]. Previous studies have identified an approximately 20-year lower life expectancy among people with intellectual disability, as well as elevated mortality from a range of causes [5], including avoidable causes of death that may be prevented through effective public health interventions or timely and effective healthcare. These inequalities represent substantial and unjust losses in years of life, and highlight persistent inequities in healthcare access, quality of care, and health outcomes experienced by people with intellectual disability and their families.

The degree of intellectual disability is generally categorised as mild, moderate, severe, or profound, based on both intelligence quotient (IQ) scores and the level of support an individual requires, as defined by the International Classification of Diseases, Tenth Revision (ICD-10) and Diagnostic and Statistical Manual of Mental Disorders, Fifth Edition (DSM-5) classification systems [1,2]. People with severe or profound intellectual disability account for approximately 5% of all individuals with intellectual disability [6]. They are more likely to have an identifiable genetic or organic cause [7], and exhibit a higher prevalence of co-occurring conditions such as constipation, epilepsy, and sensory impairment [8–11]. For example, a study conducted in the United States reported that 53% of individuals in the severe or profound group had epilepsy, compared with 24% of those with mild or moderate intellectual disability [9]. Similarly, a study from England found that 50% of individuals with severe or profound intellectual disability experienced constipation, compared with 16% of those with moderate intellectual disability and 6.5% of those with mild intellectual disability [10]. Thus, individuals with severe or profound intellectual disability often have more complex multimorbidity and may face a greater risk of premature mortality [12].

Despite a growing understanding of the prevalence and type of co-occurring conditions in people with severe or profound intellectual disability, a critical gap remains in examining important clinical outcomes. Several studies examining mortality among people with intellectual disability have been unable to stratify by level of disability, limiting insights into the specific outcomes of this group [5,13]. Moreover, the level of intellectual disability is often not treated as an independent variable in health analyses [12,14], hindering the identification of distinct risk patterns and likely underestimating the complexity of health needs among those requiring higher levels of support. Additionally, there is a lack of research examining the sociodemographic factors influencing age at death and mortality patterns in individuals with severe or profound intellectual disability, which constrains the development of targeted public health interventions. Addressing these evidence gaps is essential to reduce avoidable mortality and to support the development of equitable, evidence-based health policies and targeted interventions for people with severe or profound intellectual disability.

We therefore aimed to address three questions. First, how do the median age at death, the prevalence of co-occurring conditions, and the most common and avoidable causes of death differ among adults with severe or profound intellectual disability, adults with mild or moderate intellectual disability, and the general adult population in England? Second, how do avoidable deaths contribute to years of life lost among adults with severe or profound intellectual disability? Third, which sociodemographic factors are associated with age at death among adults with intellectual disability? We hypothesised that adults with severe or profound intellectual disability would have a higher proportion of avoidable deaths compared with the general population, and that specific factors, including level of intellectual disability and ethnicity, would be independently associated with younger age at death.

## Methods

### Study protocol

No prospective protocol or statistical analysis plan was registered for this study. The analyses reported in the original submission were specified before the data were reviewed. The study was informed by the LeDeR annual mortality report for 2023 [15], which set out the descriptive framework for mortality among adults with intellectual disability in England. Four main changes were made in response to peer review. First, we changed the age inclusion criterion for adults with intellectual disability from 18 years to 20 years, to allow direct comparison with the general population. Second, we have presented Kaplan–Meier curves to compare survival profiles across groups. As the cohort comprises only individuals who had died, there are no censored observations, and reviewers noted that a conventional time-to-event analysis is not appropriate in this setting. We therefore present the distribution of age at death and compare groups using the Kruskal–Wallis test, with Dunn's post-hoc test and Bonferroni correction for multiple comparisons. Third, we added two multiple linear regression models to examine sociodemographic factors associated with age at death, one including all adults with intellectual disability and one restricted to adults with severe or profound intellectual disability (Table 4). Fourth, we also included Cox proportional hazards models, including a model with delayed entry (left truncation) from 1 January 2021 (S5 and S6 Tables).

### Data sources

Data for this study were obtained from the English intellectual (learning) disability national mortality review programme, known as Learning from Lives and Deaths: People with a Learning Disability and Autistic People (LeDeR), a globally unique mortality dataset established in 2017 with the aim of reducing health inequalities and premature deaths among people with intellectual disability [16].

People aged 18 years or older with intellectual disability who died in England can be notified to LeDeR by a family member or friend of the deceased, carer, or health or social care professional. Notified deaths are received through the LeDeR website and then passed to the Data Services for Commissioners Regional Offices (DSCROs) for verification against the person's death record. Following verification, the deceased person's details are passed to a local reviewer who is specially trained to complete the review according to a standardised protocol. Data are entered onto an online system. The review includes information from health and social care records, interviews with people who knew the deceased in personal and professional contexts, and the Medical Certificate of Cause of Death (MCCD), which is completed by the certifying doctor at the time of death. Information collected includes sociodemographic data (e.g., date of birth, date of death, sex, ethnicity, home postcode, region of England, and place of death), clinical data (including level of intellectual disability categorised as mild, moderate, severe, or profound), and circumstances surrounding the person's death. Cause of death information was obtained from the MCCD. Reviewers also coded co-occurring physical and mental health conditions using free-text clinical information recorded within the review, including information from general practitioner records, consultation notes, and hospital discharge summaries. For this study, to enable direct comparison with the general population, we included individuals with intellectual disability aged 20 years or older, who died between January 2021 and December 2023, and whose deaths were reviewed between June 2021 and November 2025.

For comparison, aggregated mortality statistics by causes of death and individualised age at death data without personal identifiers for the general adult population in England who died in 2022 were obtained from the UK Office for National Statistics (ONS), the UK's largest independent producer of official data on population and health [17].

### Variables

We extracted sociodemographic characteristics (sex, ethnicity, home postcode, place of death, region of England, date of birth, and date of death) for each person with intellectual disability whose death underwent a LeDeR review. For the regression analysis, the outcome variable (age at death) was calculated from each individual's date of birth and date of death, with time zero defined as the participant's birth. We grouped Asian or Asian British, Black/African/Caribbean or Black British, Mixed, and Other ethnic groups into a single ‘Ethnic groups other than White ethnicity’ category because of small sample sizes. We divided England into seven regions based on National Health Service (NHS) classifications. We derived the index of multiple deprivation (IMD) from home postcode data using the Ministry of Housing, Communities and Local Government tool [18]. We grouped IMD deciles into quintiles such that 1–2 represented the most deprived areas. We extracted co-occurring physical and mental health conditions from coded fields in the LeDeR review form.

We extracted level of intellectual disability from the coded field in the review form, where reviewers drew on medical records from specialists, general practitioners, clinical psychologists, and other professionals, based on ICD-10 and DSM-5 classification systems [1,2], noting that diagnostic criteria have evolved over time with DSM-5 placing greater emphasis on adaptive functioning alongside IQ scores (details shown in S1 Table).

We identified the most common causes of death and categorised avoidable deaths according to the Organisation for Economic Co-operation and Development (OECD; S2 Table) [19]. Under this framework, avoidable deaths are those considered preventable, treatable, or both, with deaths attributed 50% to each category where both apply. Preventable causes refer to deaths that could have been avoided through primary prevention measures, such as lung cancer and vaccine-preventable infections, while treatable causes refer to deaths that could have been avoided through timely and adequate healthcare, such as epilepsy and pneumonia. The OECD framework applies to deaths occurring before the age of 75.

For the general adult population, we used ONS data on total deaths by age, sex, underlying cause of death, and area of residence in England for 2022 [20]. We obtained sex-specific life expectancy at a given age from the national life tables for England, 2021–2023 [21]. ONS datasets did not include ethnicity or co-occurring conditions data.

### Statistical analysis

We summarised sociodemographic variables—including sex, ethnicity, IMD, place of death, region of England, and age at death—for adults with severe or profound intellectual disability, mild or moderate intellectual disability, and the general adult population as counts and column percentages within each group. We used chi-squared tests to compare sociodemographic characteristics, co-occurring conditions, and the most common and avoidable causes of death between three groups. We also used the Kruskal–Wallis test to compare the age at death between three groups, post-hoc pairwise comparisons were performed using Dunn's test with Bonferroni correction.

We calculated all-causes years of life lost among adults with severe or profound intellectual disability by referring to the formula from Pan American Health Organization [22]. It was defined as follows: Σ [*D*(*c*,*s*,*a*,*t*) × SLE(*a*)], where *D*(*c*,*s*,*a*,*t*) was the number of deaths from cause *c*, sex *s*, at age *a*, in period *t*, and SLE(*a*) was the standard life expectancy remaining at specific age *a* [22]. For common causes of death and avoidable deaths, we also expressed it as a proportion of all-cause years of life lost to examine the relative contribution of specific causes to premature mortality [22].

We fitted two multiple linear regression models to examine associations between sociodemographic factors and age at death among individuals with intellectual disability. In the full cohort, we fitted four sets of models. In the first set, we estimated unadjusted effects; in the second, we adjusted for level of intellectual disability; in the third, we adjusted for ethnicity; and in the fourth, we adjusted for both level of intellectual disability and ethnicity. We additionally fitted two sets of Cox proportional hazards regression models using the same predictor variables and model structures as the multiple linear regressions. For the first set of Cox models, the outcome was age at death with no adjustment for delayed entry into the study. For the second set of Cox models, the outcome was also age at death. This second set of Cox models accounted for left truncation due to delayed study entry, since individuals were only under observation from 1 January 2021; age on 1 January 2021 was therefore used as the start of the at-risk period. We considered *p* < 0.05 statistically significant. We performed all analyses and graphing using Stata (version 19), R (version 4.4.1), and GraphPad Prism (version 10.4.1).

### Ethics statement

The LeDeR programme holds Section 251 approval from the Health Research Authority's Confidentiality Advisory Group, which granted the waiver of ethics committee approval for the processing of identifiable data without consent (Approval ref: 20/CAG/0067). Accredited researchers from King's College London, as academic partners of LeDeR, accessed de-identified mortality reviews under strict data-sharing and security protocols consistent with NHS governance standards [16,17].

### Reporting statement

This study is reported as per the Strengthening the Reporting of Observational Studies in Epidemiology (STROBE) and the Reporting of Studies Conducted using Observational Routinely-collected Data (RECORD) guideline (S1 Checklist).

### Use of Artificial Intelligence tools

Claude (Anthropic, [Opus 4.8]) was used to check grammar and clarity of the manuscript text. It was not used for study design, data collection, analysis, or interpretation, and had no access to individual-level data. The authors reviewed all suggested edits and retain full responsibility for the manuscript.

## Results

### Sociodemographic characteristics

This study included 1,301 adults with severe or profound intellectual disability and 2,626 with mild or moderate intellectual disability who died within the follow-up period. The comparison group comprised 536,311 adults aged ≥20 years from the general population (Table 1). Among adults with severe or profound intellectual disability, most were White (86.9%, *n* = 1,131), male (53.8%, *n* = 700), and died in hospital (58.8%, *n* = 765). One quarter lived in the most deprived areas (IMD quintile: 1–2: 24.9%; *n* = 324).

**Table 1:** Sociodemographic characteristics of participants by level of intellectual disability and total population comparison.

	Adults with severe or profound ID (*N* = 1,301) (%)	Adults with mild or moderate ID (*N* = 2,626) (%)	General adult population (*N* = 536,311) (%)	*p*-value (severe/profound vs mild/moderate)	*p*-value (severe/profound vs general population)
**Sex**					
Female	592 (45.5)	1,104 (42.0)	265,108 (49.4)	**0.039**	**<0.01**
Male	700 (53.8)	1,502 (57.2)	271,203 (50.6)	**0.044**	**0.020**
Not known	9 (0.7)	20 (0.8)	0 (0.0)	0.810	**<0.001**
**Ethnicity**					
Asian or Asian British	84 (6.5)	54 (2.1)	*	**<0.001**	*
Black, African, Caribbean or Black British	34 (2.6)	47 (1.8)	*	0.087	*
Mixed ethnic group	15 (1.2)	10 (0.4)	*	**<0.01**	*
Other ethnic groups	8 (0.6)	12 (0.5)	*	0.513	*
White	1,131 (86.9)	2,443 (93.0)	*	**<0.001**	*
Preferred not to say	29 (2.2)	60 (2.3)	*	0.912	*
**Index of multiple deprivation**					
1–2 (Most deprived)	324 (24.9)	729 (27.8)	108,335 (20.2)	0.057	**<0.001**
3–4	269 (20.7)	606 (23.1)	106,164 (19.8)	0.089	0.426
5–6	275 (21.1)	555 (21.1)	110,397 (20.6)	0.998	0.622
7–8	243 (18.7)	418 (15.9)	109,523 (20.4)	**0.030**	0.119
9–10 (Least deprived)	185 (14.2)	306 (11.7)	101,892 (19.0)	**0.022**	**<0.001**
Unknown	5 (0.4)	12 (0.5)	0 (0.0)	0.744	**<0.001**
**Place of death^^^**					
Hospital	765 (58.8)	1,526 (58.1)	235,588 (43.6)	0.680	**<0.001**
Care homes	229 (17.6)	482 (18.4)	110,355 (20.4)	0.564	**0.012**
Private homes	253 (19.4)	475 (18.1)	154,715 (28.6)	0.303	**<0.001**
Hospices	27 (2.1)	71 (2.7)	25,371 (4.7)	0.235	**<0.001**
Other Communal Establishments	0 (0.0)	#	1,729 (0.3)	0.223	**0.040**
Elsewhere	22 (1.7)	69 (2.6)	12,575 (2.3)	0.066	0.128
Not known	5 (0.4)	#	0 (0.0)	**<0.001**	**<0.001**
**Region of England**					
East of England	147 (11.3)	319 (12.1)	61,425 (11.5)	0.439	0.861
London	135 (10.4)	257 (9.8)	51,418 (9.6)	0.562	0.334
Midlands	302 (23.2)	534 (20.3)	108,773 (20.3)	0.038	**<0.01**
North East and Yorkshire	192 (14.8)	516 (19.6)	86,697 (16.2)	**<0.01**	0.168
North West	212 (16.3)	359 (13.7)	78,033 (14.5)	0.028	0.075
South East	205 (15.8)	402 (15.3)	87,701 (16.4)	0.714	0.562
South West	108 (8.3)	239 (9.1)	62,264 (11.6)	0.406	**<0.001**
**Age at death**					
20–24	75 (5.8)	36 (1.4)	1,246 (0.2)	**<0.001**	**<0.001**
25–49	326 (25.1)	332 (12.6)	20,846 (3.9)	**<0.001**	**<0.001**
50–64	506 (38.9)	948 (36.1)	57,484 (10.7)	0.088	**<0.001**
65+	394 (30.3)	1,310 (49.9)	456,735 (85.2)	**<0.001**	**<0.001**

Data presented in number (percentage of column total) if not specified.

ID, intellectual disability.

^*^Data on deaths by ethnic group were not available from the Office for National Statistics (ONS).

# Counts <5 are suppressed to protect confidentiality.

^ ONS place of death data for the general population also included among individuals aged <20 years. *p* values were derived using the chi-squared test.

Compared with adults with mild or moderate intellectual disability, those with severe or profound intellectual disability included a higher proportion of individuals from ethnic groups other than White ethnicity (10.8% versus 4.7%; *n* = 141 versus 123; *p* < 0.001). Compared with the general population, adults with severe or profound intellectual disability were more likely to live in the most deprived areas (24.9% versus 20.2%; *n* = 324/1,301 versus *n* = 108,335/536,311; *p* < 0.001) and a higher proportion died in hospital (58.8% versus 43.6%; *n* = 765/1,301 versus 235,588/536,311; *p* < 0.001).

### Co-occurring conditions

For those with reviewer-coded data, adults with severe or profound intellectual disability had significantly higher recorded rates of dysphagia (63.0%; *n* = 613/973), epilepsy (36.1%; *n* = 351) and visual problems (35.9%; *n* = 349) than those with mild or moderate intellectual disability (*p* < 0.01 for each) (Table 2). Conversely, kidney problems, hypertension, diabetes and cancer were more common in adults with mild or moderate intellectual disability. For mental health conditions, anxiety, depression and psychosis were more common in adults with mild or moderate intellectual disability (*p* < 0.01 for each) (Table 2).

**Table 2:** Percentage and type of co-occurring health conditions by level of intellectual disability and disease categories.

Long-term conditions	Adults with severe or profound ID (*N* = 973) (%)	Adults with mild or moderate ID (*N* = 1,933) (%)	*p*-value
**Physical health conditions**			
Dysphagia	613 (63.0)	756 (39.1)	**<0.001**
Epilepsy	351 (36.1)	417 (21.6)	**<0.001**
Visual problems	349 (35.9)	579 (30.0)	**<0.01**
Kidney problems	189 (19.4)	519 (26.8)	**<0.001**
Dementia	146 (15.0)	344 (17.8)	0.058
Hypertension	137 (14.1)	539 (27.9)	**<0.001**
Hearing problems	130 (13.4)	345 (17.8)	**<0.01**
Diabetes	108 (11.1)	512 (26.5)	**<0.001**
Asthma	101 (10.4)	261 (13.5)	**0.016**
Thyroid disorder	89 (9.1)	230 (11.9)	**0.025**
Cancer	83 (8.5)	315 (16.3)	**<0.001**
Stroke	56 (5.8)	148 (7.7)	0.058
Osteoporosis	36 (3.7)	58 (3.0)	0.315
Deep vein thrombosis	28 (2.9)	103 (5.3)	**<0.01**
Chronic obstructive pulmonary disease	18 (1.8)	158 (8.2)	**<0.001**
Coronary artery disease	*	56 (2.9)	**<0.001**
Peripheral artery disease	*	18 (0.9)	0.061
**Mental health conditions**			
Anxiety	241 (24.8)	588 (30.4)	**<0.01**
Depression	101 (10.4)	469 (24.3)	**<0.001**
Bipolar	39 (4.0)	82 (4.2)	0.766
Psychosis	38 (3.9)	252 (13.0)	**<0.001**

Data are presented as numbers (percentages of adults in each group) if not specified. ID = intellectual disability.

*Suppressed for those ≤5 cases. *p* values were derived using the chi-squared test.

### Age at death, causes of death, and years of life lost

**Fig 1:**
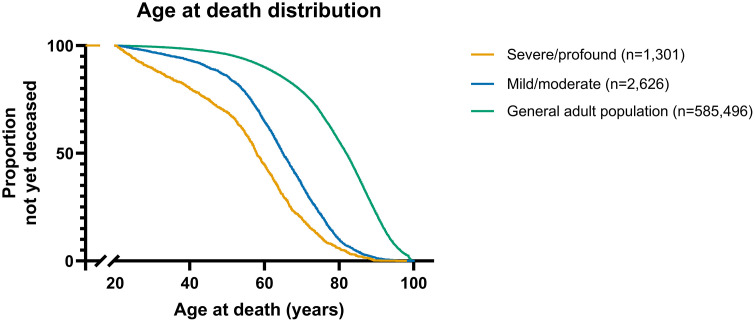
Age at death distribution by level of intellectual disability compared with total adult population. Adults with severe or profound intellectual disability (n = 1,301) had a significantly lower median age at death, compared to those with mild or moderate intellectual disability (*n* = 2,626) (57.9 years vs 65.0 years) and the general population (*n* = 585,496) (57.9 years vs 81.9 years) (both *p* < 0.001). Severe or profound = Adults with severe or profound intellectual disability; mild or moderate = Adults with mild or moderate intellectual disability. *General adult population involved data from England and Wales from Office for National Statistics. *p* values were derived using the Kruskal–Wallis test with Dunn's post-hoc test and Bonferroni correction.

Overall, adults with severe or profound intellectual disability had a significantly lower median age at death compared to those with mild or moderate intellectual disability (57.9 years versus 65.0 years) and the general population (57.9 years versus 81.9 years) (both *p* < 0.001) (Fig 1). Adults with severe or profound intellectual disability showed a steeper rate of death beginning around age 20 compared with adults with mild or moderate intellectual disability and the general population, indicating a higher probability of early death (Fig 1).

Among individuals aged 20–24 and 25–49 years with severe or profound intellectual disability, the leading cause of death was diseases of the nervous system, accounting for 38.7% (*n* = 29/75) and 36.2% (*n* = 118/326) of deaths, respectively (S3 Table). The proportion of deaths due to nervous system diseases in the 25–49 age group was significantly higher than in both adults with mild or moderate intellectual disability and the general population (*p* < 0.001 each). After age 49, the cumulative mortality increased sharply, indicating a greater concentration of deaths at older ages. The most common causes of death among those aged 50–64 years were congenital malformations, deformations, and chromosomal abnormalities (21.3%; *n* = 108/506), nervous system diseases (18.0%; *n* = 91) and respiratory system diseases (17.6%; *n* = 89). We also observed significantly higher proportions of deaths from diseases of the nervous system and respiratory system, compared with both the mild or moderate intellectual disability group and the general population (all *p* < 0.01). From age 65 onwards, the age at death curve began to plateau, with respiratory system diseases accounting for the highest proportion of deaths in those who died over the age of 65 years (22.8%; *n* = 90/394) (S3 Table).

Across all ages, the leading causes of death among adults with severe or profound intellectual disability were diseases of the nervous system (22.5%; *n* = 293/1,301), respiratory system (16.6%; *n* = 216), and congenital malformations, deformations, and chromosomal abnormalities (14.8%; *n* = 193) (S1 Fig). The proportions of diseases of the nervous system and congenital malformations, deformations, and chromosomal abnormalities were also significantly higher than in adults with mild or moderate intellectual disability and the general population (*p* < 0.01 for each). One-tenth of deaths were due to neoplasms (10.7%; *n* = 139) or circulatory diseases (10.3%; *n* = 134). The 1,301 deaths in this group corresponded to 37,044.7 years of life lost, equivalent to 28.5 years per person (S4 Table).

### Avoidable mortality

Two-fifths of deaths among adults with severe or profound intellectual disability were classified as avoidable (39.5%; *n* = 514/1,301) (Table 3), resulting in 15,059.4 years of life lost, or 40.7% of the all-cause years lost (S4 Table). The percentage of avoidable deaths was slightly lower than in adults with mild or moderate intellectual disability (39.5% versus 42.7%; *n* = 514/1,301 versus *n* = 1,121/2,626; *p* = 0.057), while being significantly higher than in the general population (39.5% versus 21.8%; *n* = 514/1,301 versus *n* = 116,701/536,311; *p* < 0.001).

**Table 3:** Percentage and most common causes of avoidable mortality by level of intellectual disability and disease categories.

	Adults with severe or profound ID (*N* = 1,301) (%)	Adults with mild or moderate ID (*N* = 2,626) (%)	General adult population (*N* = 536,311) (%)	*p*-value (severe/profound vs mild/moderate)	*p*-value (severe/profound vs general population)
**Category of avoidable mortality**					
Avoidable	514 (39.5)	1,121 (42.7)	116,701 (21.8)	0.057	**<0.001**
Preventable	162 (12.5)	486.5 (18.5)	40,610.5 (7.6)	**<0.001**	**<0.001**
Treatable	352 (27.1)	634.5 (24.2)	76,090.5 (14.2)	**0.049**	**<0.001**
**Most common causes of avoidable mortality (ICD-10 codes)**					
Pneumonia, not elsewhere classified or organism unspecified	87 (6.7)	126 (4.8)	3,166 (0.6)	**0.014**	**<0.001**
Epilepsy	64 (4.9)	58 (2.2)	560 (0.1)	**<0.001**	**<0.001**
Cerebrovascular diseases	43 (3.3)	74 (2.8)	6,128 (1.1)	0.398	**<0.001**
COVID-19	32 (2.5)	51 (1.9)	21,025 (3.9)	0.289	**<0.01**
Lung diseases due to external agents	30 (2.3)	22 (0.8)	13,001 (2.4)	**<0.001**	0.782
Ischaemic heart diseases	29 (2.2)	127 (4.8)	20,554 (3.8)	**<0.001**	**<0.01**

Data are presented as numbers (percentages of adults in each group if not specified). ID, intellectual disability. ICD-10, International Classification of Diseases, Tenth Revision. *p* values were derived using the chi-squared test.

**Table 4:** Multiple linear regression of factors associated with age at death among adults with intellectual disability.

Factors	Unadjusted mean difference (years) (95% CI)	*p*-value	Level of ID adjusted mean difference (years) (95% CI)	*p*-value	Ethnicity adjusted mean difference (years) (95% CI)	*p*-value	Level of ID and ethnicity adjusted mean difference (years) (95% CI)	*p*-value
**All adults with ID (*N* = 3,927)**								
**Level of ID**								
Mild or moderate (reference)	–	–	–	–			–	–
Severe or profound	**−8.06** **(−9.05, −7.06)**	**<0.001**	**–**	**–**	**−7.17** **(−8.16, −6.18)**	**<0.001**	**–**	**–**
**Ethnicity**								
White (reference)	–	–	–	–	–	–	**–**	**–**
Ethnic groups other than White ethnicity	**−14.94** **(−16.82, −13.07)**	**<0.001**	**−13.38** **(−15.22, −11.54)**	**<0.001**	**–**	**–**	–	–
**Sex**								
Female (reference)	–	–	–	–	–	–	–	–
Male	0.03 (−0.95, 1.01)	0.951	−0.22 (−1.17, 0.73)	0.648	0.42 (−0.54, 1.38)	0.395	0.17 (−0.77, 1.11)	0.722
**Index of multiple deprivation (IMD)**								
9–10 (least deprived, reference)	–	–	–	–	–	–	–	–
7–8	−0.06 (−1.87, 1.75)	0.949	−1.13 (−1.88, 1.62)	0.881	−0.04 (−1.82, 1.73)	0.963	−0.13 (−1.86, 1.60)	0.882
5–6	−0.58 (−2.31, 1.14)	0.508	−0.95 (−2.63, 0.72)	0.265	−0.57 (−2.26, 1.12)	0.509	−0.94 (−2.59, 0.71)	0.265
3–4	0.14 (−1.57, 1.85)	0.874	−0.42 (−2.08, 1.24)	0.616	0.57 (−1.11, 2.24)	0.507	0.03 (−1.60, 1.67)	0.968
1–2 (most deprived)	−0.84 (−2.5, 0.82)	0.319	−1.40 (−3.01, 0.21)	0.087	−0.36 (−1.99, 1.27)	0.664	−0.90 (−2.49, 0.69)	0.265
**Adults with severe or profound ID (N = 1,301)**								
**Ethnicity**								
White (reference)	–	–	–	–	–	–	–	–
Ethnic groups other than White ethnicity	**−14.28** **(−17.10, −11.45)**	**<0.001**	–	–	–	–	–	–
**Sex**								
Female (reference)	–	–	–	–	–	–	–	–
Male	−0.37 (−2.19, 1.45)	0.691	–	–	0.19 (−1.61, 1.98)	0.840	–	–
**Index of multiple deprivation (IMD)**								
9–10 (least deprived, reference)	–	–	–	–	–	–	–	–
7–8	0.61 (−2.58, 3.80)	0.708	–	–	0.71 (−2.41, 3.83)	0.655	–	–
5–6	−1.75 (−4.86, 1.35)	0.269	–	–	-1.61 (−4.65, 1.43)	0.299	–	–
3–4	0.15 (−2.97, 3.27)	0.926	–	–	0.97 (−2.06, 4.01)	0.530	–	–
1–2 (most deprived)	−0.18 (−3.19, 2.83)	0.908	–	–	1.00 (−1.95, 3.95)	0.505	–	–

CI, confidence interval; ID, intellectual disability; IMD, index of multiple deprivation. *p* values were derived using multiple linear regression.

The proportion of deaths due to preventable causes was lower in adults with severe or profound intellectual disability than in adults with mild or moderate intellectual disability (12.5% versus 18.5%; *n* = 162/1,301 versus *n* = 486.5/2,626; *p* < 0.001), but significantly higher than in the general population (12.5% versus 7.6%; *n* = 162/1,301 versus *n* = 40,610.5/536,311; *p* < 0.001). Conversely, the proportion of deaths due to treatable causes was higher in adults with severe or profound intellectual disability compared with those with mild or moderate intellectual disability (27.1% versus 24.2%; *n* = 352/1,301 versus *n* = 634.5/2,626; *p* = 0.049), as well as the general population (27.1% versus 14.2%; or *n* = 352/1,301 versus *n* = 76,090.5/536,311; *p* < 0.001).

The leading avoidable causes of death among adults with severe or profound intellectual disability were pneumonia, not elsewhere classified or organism unspecified (classified as preventable and treatable; 6.7% of all deaths, *n* = 87), epilepsy (treatable; 4.9%; *n* = 64), and cerebrovascular diseases (both preventable and treatable; 3.3%; *n* = 43). Both pneumonia and epilepsy were significantly more common than in adults with mild or moderate intellectual disability and the general population (*p* < 0.05).

### Factors associated with age at death

In the multiple linear regression model including all adults with intellectual disability who died during the follow-up period (Table 4), the following were associated with a younger age at death: having a severe or profound intellectual disability (adjusted mean difference −7.17 years; 95% confidence interval [CI] [−8.16, − 6.18]; *p* < 0.001) and being from an ethnic group other than White (adjusted mean difference −13.38 years; 95% CI [−15.22, − 11.54]; *p* < 0.001). The sensitivity analyses using Cox proportional hazards regression are presented in S5 and S6 Tables. Adjusting for ethnicity, there was statistical evidence of an association between having a severe or profound intellectual disability and hazard of death within this death cohort (adjusted hazard ratio [HR] 1.47; 95% CI [1.37, 1.57]; *p* < 0.001). With delayed entry, there was no statistical evidence of an association (adjusted HR 1.04; 95% CI [0.97, 1.11]; *p* = 0.316).

A model restricted to adults with severe or profound intellectual disability (Table 4) also showed that being from an ethnic group other than White was associated with a younger age at death (unadjusted mean difference −14.28 years; 95% CI [−17.10, −11.45]; *p* < 0.001).

## Discussion

This population-based cohort study highlights the differing clinical profiles and younger age at death among people with severe or profound intellectual disability compared with those with mild or moderate intellectual disability. It also demonstrates striking disparities in avoidable mortality rates in both groups when compared with the general population. These findings represent a major public health concern and warrant focused attention from practitioners and health systems worldwide [23,24]. Although several local and national initiatives have been implemented across health and social care sectors in the UK over recent years, including intellectual disability registers and annual health checks in primary care, care services and outcomes for people with intellectual disability must be further improved [15,25]. As people with intellectual disability have nearly twice as many general practice consultations as those without intellectual disability [26], primary care will remain central to ongoing efforts to reduce health inequities in this population.

This is the first study to compare co-occurring physical and mental health profiles of adults with severe or profound intellectual disability with those of adults with mild or moderate disability, revealing distinct patterns across groups. Epilepsy, dysphagia, and visual problems were the most common physical conditions reported in adults with severe or profound intellectual disability, consistent with previous studies [8,27]. In contrast, age-related long-term conditions such as hypertension, kidney problems and diabetes were more prevalent in adults with mild or moderate intellectual disability, possibly reflecting their longer life span and consequently greater chance to develop age-associated comorbidities [28–30]. Clinicians should be aware of these differing needs and adapt healthcare strategies accordingly. Adults with severe or profound intellectual disability often require specialist epilepsy management, particularly since epilepsy in this group is more likely to have a genetic origin and be resistant to standard treatments [31]. Dysphagia also demands careful clinical assessment and clear guidance for carers to prevent complications such as aspiration pneumonia, a frequent cause of death in this population [32]. In contrast, adults with mild or moderate intellectual disability may benefit most from accessible health promotion programmes focusing on lifestyle behaviours, diet and physical activity levels to reduce long-term conditions, metabolic syndrome and their sequelae [33]. The introduction of a specially trained intellectual disability physician, a role well established in the Netherlands, has been proposed to address these complex needs [34,35]. Such a model could be prioritised for those with severe or profound intellectual disability. Assigning each person with intellectual disability a dedicated care coordinator (e.g., a practice nurse) has also been recommended [15], and could improve timely access to healthcare by ensuring that people with multiple chronic conditions are able to navigate the health system with the necessary reasonable adjustments in place.

The lower recorded prevalence of specific mental health conditions among adults with severe or profound intellectual disability mirrors findings from previous studies [36]. The true prevalence is likely underestimated, owing to diagnostic challenges linked to communication and cognitive impairments, as well as to diagnostic overshadowing, whereby symptoms are attributed to the intellectual disability rather than to a separate, potentially treatable condition [7]. The absence of validated assessment tools and the poor applicability of standard diagnostic criteria further complicate accurate diagnosis in this group [37,38]. Efforts to mitigate diagnostic overshadowing in England include the introduction of annual health checks and the NHS reasonable adjustment flag to improve recognition of health conditions in people with intellectual disability [39,40]. Nevertheless, robust quantitative estimates of the proportion of avoidable deaths attributable to diagnostic overshadowing remain scarce, with existing evidence largely qualitative or descriptive in nature [41]. Future studies are needed to evaluate the effectiveness of these initiatives in improving mental health recognition and diagnosis among people with intellectual disability.

Our study demonstrates a distinct age-at-death pattern among adults with severe or profound intellectual disability, with an average age at death over seven years younger than those with mild or moderate intellectual disability and approximately 25 years younger than the general population. This persistent mortality gap aligns with global evidence [14,42,43]. Our data reveal that adults with severe or profound intellectual disability experience a sustained increase in cumulative mortality from early adulthood onwards, whereas those with mild or moderate disability show an age-at-death pattern closer to that of the general population, with a steeper increase emerging after age 50. Diseases of the nervous system and congenital or chromosomal abnormalities accounted for nearly half of all deaths before age 50 in the severe or profound group—substantially higher than in those with mild or moderate disability. This is consistent with the higher likelihood of genetic or syndromic aetiologies among people with severe or profound intellectual disability [44], many of which are associated with progressive or life-limiting comorbidities such as neurocutaneous syndromes and metabolic disorders [45].

To our knowledge, this is the first study to show that two in five deaths among adults with severe or profound intellectual disability are due to avoidable causes, a significantly higher percentage compared with the general population, although slightly lower than that among adults with mild or moderate intellectual disability. This aligns with population-based findings from Scotland that 31.7% of deaths among adults with intellectual disability (of any degree) between 2011 and 2019 were from avoidable causes, compared with 18.7% in the general population [13]. Within avoidable causes of death, preventable mortality was more common in those with mild or moderate intellectual disability whereas treatable mortality was more common in those with severe or profound intellectual disability. This suggests that public health campaigns, including vaccination and cancer screening, are not effectively reaching people with mild or moderate intellectual disability [46,47]. The higher proportion of treatable causes among adults with severe or profound intellectual disability implies delayed recognition and late presentation of acute illness (e.g., appendicitis, acute respiratory infections) and barriers to accessing appropriate treatment, including perhaps under-treatment and a ‘ceiling of care’ imposed on the basis of assumptions about disability and quality of life [48]. Of note, avoidable mortality is classified at the condition level based on OECD criteria. This does not imply that every individual who died from these conditions received inadequate care. An individual person may receive screening, vaccination or treatment and still die from a cause classified as avoidable. However, the disproportionately high rate of avoidable deaths among adults with intellectual disability reflects a population-level signal of greater vulnerability and health inequalities compared with the general population.

We quantified avoidable mortality in terms of years of life lost and examined factors associated with age at death. Severe or profound intellectual disability was strongly associated with younger death, consistent with previous studies [14,43]. This analysis also revealed that individuals from ethnic minority groups with severe or profound intellectual disability died at a significantly younger age compared with people of White ethnicity in England. This finding may highlight additional challenges in accessing healthcare encountered by people from ethnic minority groups, reflecting intersectional inequalities related to disability, health, and social determinants of health [49].

This study used a large, national dataset containing comprehensive sociodemographic and clinical information on adults with intellectual disability across England. A key strength is the stratification by level of intellectual disability from various medical sources, which most previous studies could not achieve [5,13]. The robustness of the dataset allowed us to characterise patterns in co-occurring health conditions, age at death and causes of death across different levels of intellectual disability. Our findings address an important evidence gap for this under-represented population, which is often excluded from clinical research [50,51].

This study has limitations. First, there is a risk of sampling bias. Although reporting a death to LeDeR is strongly encouraged and includes robust NHS systems to ensure timely reporting, it is not mandatory and it is possible that some deaths in England may have gone unreported. Although extensive public promotion of the LeDeR programme has been associated with an increase in notifications of deaths across England and improved population coverage, as evidenced by rising reported cases between 2021 and 2023 [15], some groups, particularly those from ethnic minority backgrounds, may remain under-represented [49]. Second, because LeDeR data include only deceased individuals, they provides no information on people with intellectual disability who are alive, including their health needs and experiences. This restricts our ability to assess age-related trends within subgroups, particularly among ethnic minority groups. Therefore, findings related to age at death by ethnicity should be interpreted with caution. For instance, if the population of adults with intellectual disabilities from ethnic minority backgrounds is younger on average than their White counterparts, this may have influenced the results of regression models. Third, missing data on cause of death may have influenced estimates of avoidable mortality, although this limitation likely affected all groups similarly. Fourth, the general population mortality data from the ONS also include deaths among people with intellectual disability, which may lead to an under-estimation of differences in causes of death between the two populations. Fifth, as the LeDeR and ONS mortality datasets could not be linked at the individual level, we were unable to conduct a combined analysis comparing avoidable mortality between adults with intellectual disability and the general population in a single regression model after adjusting for covariates. Sixth, multiple linear regression does not account for delayed study entry arising from individuals only being observable from 1 January 2021 [52,53]. Using a Cox model with delayed entry, we found smaller effect sizes. These models assume that event time is independent of entry time conditional on covariates, an assumption which may have been broken if there was informative left truncation due to differential survival to observation (for example due to possible COVID-era mortality shifts or potentially differential selection of individuals). Finally, our analysis did not include children or adolescents, limiting generalisability to younger populations.

This large-scale, population-based study shows that adults with severe or profound intellectual disability die at significantly younger ages than those with mild or moderate intellectual disability, and that both groups experience much higher rates of avoidable deaths than the general population. The differing patterns of underlying co-occurring conditions and causes of death suggest the need for tailored strategies to address health inequities within this population. Adults with mild or moderate intellectual disability may benefit most from accessible health promotion initiatives targeting preventable conditions such as diabetes, hypertension, and obesity, whereas those with severe or profound intellectual disability require focused interventions such as specialist epilepsy management and vaccination against respiratory infections. The additional disparities observed among people from ethnic minority backgrounds warrant urgent investigation. Primary care must play a central role in prevention (e.g., access to screening and vaccinations) to detect and address disease in its early stages. The development of new professional roles dedicated to the care of people with intellectual disability and the use of routinely collected health data to monitor outcomes and evaluate system-level interventions, such as the NHS reasonable adjustment digital flag, could further improve health equity and outcomes in this vulnerable population. Furthermore, future studies comparing the prevalence of co-occurring conditions between people with severe or profound intellectual disability and the general population, as well as the independent association between intellectual disability severity and avoidable mortality, could help us better understand the unique health needs of this group.

## Supporting information

S1 Table Classification of intellectual disability severity by ICD-10 and DSM-5 criteria. (DOCX)

S2 Table List of avoidable deaths as defined by the Organisation for Economic Co-operation and Development (OECD). (DOCX)

S3 Table Percentage and breakdown of mortality by level of intellectual disability, disease categories and age group. (DOCX)

S4 Table Years of life lost in adults with severe or profound intellectual disability by disease category (ICD-10 chapters and OECD definition) and sex. (DOCX)

S5 Table Cox regression predicting age at death among adults with intellectual disability. (DOCX)

S6 Table Cox regression predicting age at death among adults with intellectual disability and delayed entry (left truncation) at 1 January 2021. (DOCX)

S1 Fig Most common causes of death by level of intellectual disability. Among adults with severe or profound intellectual disability (*n* = 1,301), the most common causes of death were diseases of the nervous system (22.5%, *n* = 293), diseases of the respiratory system (16.6%, *n* = 216), and congenital malformations, deformations, and chromosomal abnormalities (14.8%, *n* = 193). Compared with adults with mild or moderate intellectual disability, a higher proportion of adults with severe or profound intellectual disability died from diseases of the nervous system and from congenital malformations, deformations, and chromosomal abnormalities (*p* < 0.01 for each comparison). Additionally, around one-tenth died from neoplasms (10.7%, *n* = 139) or diseases of the circulatory system (10.3%, *n* = 134). p values were derived using the chi-squared test. Severe or profound = Adults with severe or profound intellectual disability; mild or moderate = Adults with mild or moderate intellectual disability.
(TIF)

S1 Checklist STROBE and RECORD checklist. *Reference: Benchimol EI, Smeeth L, Guttmann A, Harron K, Moher D, Petersen I, Sørensen HT, von Elm E, Langan SM, the RECORD Working Committee. The REporting of studies Conducted using Observational Routinely-collected health Data (RECORD) Statement. *PLoS Medicine* 2015; in press. *Checklist is protected under Creative Commons Attribution (CC BY) license.
(DOCX)

## Abbreviations

CI: confidence interval
DSM-5: Diagnostic and Statistical Manual of Mental Disorders, Fifth Edition
HR: hazard ratio
ICD-10: International Classification of Diseases, Tenth Revision
IMD: index of multiple deprivation
IQ: intelligence quotient
LeDeR: Learning from Lives and Deaths: People with a Learning Disability and Autistic People
NHS: National Health Service
OECD: Organisation for Economic Co-operation and Development
ONS: Office for National Statistics
