# Supplementary material for: Avoidable mortality, clinical outcomes and determinants of premature death among adults with severe or profound intellectual disability: A population-based analysis of mortality data in England
Source: PLoS Med. 2026 Aug 17;23(8):e1005029. doi: 10.1371/journal.pmed.1005029 (PMC13480647; doi:10.1371/journal.pmed.1005029)
Supplement: Supplementary file 1 [file pmed.1005029.s001.docx]

Supplementary Table 1: Classification of intellectual disability severity by ICD-10 and DSM-5 criteria

| Severity | IQ Range (ICD-10) | Adaptive Functioning (DSM-5) | Level of Support required |
| --- | --- | --- | --- |
| Mild | 50–69 | Difficulties in academic learning; concrete thinking; age-appropriate self-care largely possible; benefits from vocational training | Intermittent. support needed in specific situations (e.g. finances, healthcare navigation) |
| Moderate | 35–49 | Marked delays in development; limited academic achievement; communication possible but limited in complexity; benefits from structured routines | Limited/consistent. Regular support across most daily activities; supervised living arrangements |
| Severe | 20–34 | Considerable motor impairment; very limited academic skills; may use augmentative and alternative communication (e.g. symbols, signs); understanding of speech limited | Extensive. Continuous support across most life domains; specialist health monitoring required |
| Profound | Below 20 | Severely limited or absent verbal communication; significant physical and sensory impairments commonly co-occur; highly dependent on others for all activities | Pervasive. 24-hour specialist care across all life domains; assistive technology frequently required |

IQ = intelligence quotient; ICD-10 = International Classification of Diseases, 10th Revision; DSM-5 = Diagnostic and Statistical Manual of Mental Disorders, 5th Edition. Source: World Health Organization and American Psychiatric Association.[1, 2]

References:

1.	WHO. The ICD-10 classification of mental and behavioural disorders: World Health Organization; 1992.

2.	Diagnostic and statistical manual of mental disorders, Fifth Edition. American Psychiatric Association. 2013;21(21):591-643.
