# Supplementary material for: Avoidable mortality, clinical outcomes and determinants of premature death among adults with severe or profound intellectual disability: A population-based analysis of mortality data in England
Source: PLoS Med. 2026 Aug 17;23(8):e1005029. doi: 10.1371/journal.pmed.1005029 (PMC13480647; doi:10.1371/journal.pmed.1005029)
Supplement: Supplementary file 2 [file pmed.1005029.s002.docx]

# Supplementary Table 2: List of avoidable deaths as defined by the Organisation for Economic Co-operation and Development (OECD)

| **Condition group and cause** | **ICD-10 codes** | **Age** | **Treatable** | **Preventable** |
| --- | --- | --- | --- | --- |
| **Infectious diseases** | | | | |
| Intestinal diseases | A00-A09 | 0-74 |  | • |
| Diphtheria, Tetanus, Poliomyelitis | A35, A36, A80 | 0-74 |  | • |
| Whooping cough | A37 | 0-74 |  | • |
| Meningococcal infection | A39 | 0-74 |  | • |
| Sepsis due to streptococcus pneumonia and sepsis due to haemophilus influenzae | A40.3, A41.3 | 0-74 |  | • |
| Haemophilus influenza infections | A49.2 | 0-74 |  | • |
| Sexually transmitted infections (except HIV/AIDS) | A50-A60, A63, A64 | 0-74 |  | • |
| Varicella | B01 | 0-74 |  | • |
| Measles | B05 | 0-74 |  | • |
| Rubella | B06 | 0-74 |  | • |
| Viral Hepatitis | B15-B19 | 0-74 |  | • |
| HIV/AIDS | B20-B24 | 0-74 |  | • |
| Malaria | B50-B54 | 0-74 |  | • |
| Haemophilus and pneumococcal meningitis | G00.0, G00.1 | 0-74 |  | • |
| Tuberculosis | A15-A19, B90, J65 | 0-74 | • (50%) | • (50%) |
| Scarlet fever | A38 | 0-74 | • |  |
| Sepsis | A40 (excl. A40.3), A41 (excl. A41.3) | 0-74 | • |  |
| Cellulitis | A46, L03 | 0-74 | • |  |
| Legionnaires disease | A48.1 | 0-74 | • |  |
| Streptococcal and enterococci infection | A49.1 | 0-74 | • |  |
| Other meningitis | G00.2, G00.3, G00.8, G00.9 | 0-74 | • |  |
| Meningitis due to other and unspecified causes | G03 | 0-74 | • |  |
| **Neoplasms** |  | 0-74 |  |  |
| Lip, oral cavity and pharynx cancer | C00-C14 | 0-74 |  | • |
| Oesophageal cancer | C15 | 0-74 |  | • |
| Stomach cancer | C16 | 0-74 |  | • |
| Liver cancer | C22 | 0-74 |  | • |
| Lung cancer | C33-C34 | 0-74 |  | • |
| Mesothelioma | C45 | 0-74 |  | • |
| Skin (melanoma) cancer | C43 | 0-74 |  | • |
| Bladder cancer | C67 | 0-74 |  | • |
| Cervical cancer | C53 | 0-74 | • (50%) | • (50%) |
| Colorectal cancer | C18-C21 | 0-74 | • |  |
| Breast cancer (female only) | C50 | 0-74 | • |  |
| Uterus cancer | C54, C55 | 0-74 | • |  |
| Testicular cancer | C62 | 0-74 | • |  |
| Thyroid cancer | C73 | 0-74 | • |  |
| Hodgkin's disease | C81 | 0-74 | • |  |
| Lymphoid leukaemia | C91.0, C91.1 | 0-74 | • |  |
| Benign neoplasm | D10-D36 | 0-74 | • |  |
| **Endocrine and metabolic diseases** | | | | |
| Nutritional deficiency anaemia | D50-D53 | 0-74 |  | • |
| Diabetes mellitus | E10-E14 | 0-74 | • (50%) | • (50%) |
| Thyroid diseases | E00-E07 | 0-74 | • |  |
| Adrenal diseases | E24-E25 (excl. E24.4), E27 | 0-74 | • |  |
| **Diseases of the nervous system** | | | | |
| Epilepsy | G40, G41 | 0-74 | • |  |
| **Diseases of the circulatory system** | | | | |
| Aortic aneurysm | I71 | 0-74 | • (50%) | • (50%) |
| Hypertensive diseases | I10-I13, I15 | 0-74 | • (50%) | • (50%) |
| Ischaemic heart diseases | I20-I25 | 0-74 | • (50%) | • (50%) |
| Cerebrovascular diseases | I60-I69 | 0-74 | • (50%) | • (50%) |
| Other atherosclerosis | I70, I73.9 | 0-74 | • (50%) | • (50%) |
| Rheumatic and other heart diseases | I00-I09 | 0-74 | • |  |
| Venous thromboembolism | I26, I80, I82.9 | 0-74 | • |  |
| **Diseases of the respiratory system** | | | | |
| Influenza | J09-J11 | 0-74 |  | • |
| Pneumonia due to streptococcus pneumonia or haemophilus influenza | J13-J14 | 0-74 |  | • |
| Chronic lower respiratory diseases | J40-J44 | 0-74 |  | • |
| Lung diseases due to external agents | J60-J64, J66-J70, J82, J92 | 0-74 |  | • |
| Upper respiratory infections | J00-J06, J30-J39 | 0-74 | • |  |
| Pneumonia, not elsewhere classified or organism unspecified | J12, J15, J16-J18 | 0-74 | • |  |
| Acute lower respiratory infections | J20-J22 | 0-74 | • |  |
| Asthma and bronchiectasis | J45-J47 | 0-74 | • |  |
| Adult respiratory distress syndrome | J80 | 0-74 | • |  |
| Pulmonary oedema | J81 | 0-74 | • |  |
| Abscess of lung and mediastinum pyothorax | J85, J86 | 0-74 | • |  |
| Other pleural diseases | J90, J93, J94 | 0-74 | • |  |
| **Diseases of the digestive system** | | | | |
| Gastric and duodenal ulcer | K25-K28 | 0-74 | • |  |
| Appendicitis | K35-K38 | 0-74 | • |  |
| Abdominal hernia | K40-K46 | 0-74 | • |  |
| Cholelithiasis and cholecystitis | K80-K81 | 0-74 | • |  |
| Other diseases of gallbladder or biliary tract | K82-K83 | 0-74 | • |  |
| Acute pancreatitis | K85.0, K85.1, K85.3, K85.8, K85.9 | 0-74 | • |  |
| Other diseases of pancreas | K86.1, K86.2, K86.3, K86.8, K86.9 | 0-74 | • |  |
| **Diseases of the genitourinary system** | | | | |
| Nephritis and nephrosis | N00-N07 | 0-74 | • |  |
| Obstructive uropathy | N13, N20-N21, N35 | 0-74 | • |  |
| Renal failure | N17-N19 | 0-74 | • |  |
| Renal colic | N23 | 0-74 | • |  |
| Diseases resulting from renal tubular dysfunction | N25 | 0-74 | • |  |
| Unspecified contracted kidney, small kidney of unknown cause | N26-N27 | 0-74 | • |  |
| Inflammatory diseases of genitourinary system | N34.1, N70-N73, N75.0, N75.1, N76.4, N76.6 | 0-74 | • |  |
| Prostatic hyperplasia | N40 | 0-74 | • |  |
| **Pregnancy, childbirth and the perinatal period** | | | | |
| Tetanus neonatorum | A33 | 0-74 |  | • |
| Obstetrical tetanus | A34 | 0-74 |  | • |
| Pregnancy, childbirth and the puerperium | O00-O99 | 0-74 | • |  |
| Certain conditions originating in the perinatal period | P00-P96 | 0-74 | • |  |
| **Congenital malformations** | | | | |
| Certain congenital malformations (neural tube defects) | Q00, Q01, Q05 | 0-74 |  | • |
| Congenital malformations of the circulatory system (heart defects) | Q20-Q28 | 0-74 | • |  |
| Adverse effects of medical and surgical care |  | 0-74 |  |  |
| Drugs, medicaments and biological substances causing adverse effects in therapeutic use | Y40-Y59 | 0-74 | • |  |
| Misadventures to patients during surgical and medical care | Y60-Y69, Y83-Y84 | 0-74 | • |  |
| Medical devices associated with adverse incidents in diagnostic and therapeutic use | Y70–Y82 | 0-74 | • |  |
| **Injuries** |  |  |  |  |
| Transport Accidents | V01-V99 | 0-74 |  | • |
| Accidental Injuries | W00-X39, X46-X59 | 0-74 |  | • |
| Intentional self-harm | X66-X84 | 0-74 |  | • |
| Event of undetermined intent | Y16-Y34 | 0-74 |  | • |
| Assault | X86-Y09 | 0-74 |  | • |
| **Alcohol-related and drug-related deaths** | | | | |
| Alcohol-specific diseases and poisonings | E24.4, F10, G31.2, G62.1, G72.1, I42.6, K29.2, K70, K85.2, K86.0, Q86.0, R78.0, X45, X65, Y15 | 0-74 |  | • |
| Other alcohol-related diseases | K73, K74.0-K74.2, K74.6 | 0-74 |  | • |
| Drug diseases and poisonings | F11-F16, F18-F19, X40-X44, X85, Y10-Y14 | 0-74 |  | • |
| Intentional self-poisoning by drugs | X60-X64 | 0-74 |  | • |
| **Provisional assignment of new diseases** | | | | |
| COVID-19 | U07.1-U07.2 | 0-74 |  | • |

Source: Organisation for Economic Co-operation and Development [1]. ICD-10 = International Classification of Diseases, 10th Revision.

References:

1.	Avoidable mortality: OECD/Eurostat lists of preventable and treatable causes of death (January 2022 version): Organisation for Economic Cooperation and Development; 2022 [Available from: <https://www.oecd.org/content/dam/oecd/en/data/datasets/oecd-health-statistics/avoidable-mortality-2019-joint-oecd-eurostat-list-preventable-treatable-causes-of-death.pdf>.
