# Supplementary material for: Avoidable mortality, clinical outcomes and determinants of premature death among adults with severe or profound intellectual disability: A population-based analysis of mortality data in England
Source: PLoS Med. 2026 Aug 17;23(8):e1005029. doi: 10.1371/journal.pmed.1005029 (PMC13480647; doi:10.1371/journal.pmed.1005029)
Supplement: Supplementary file 3 [file pmed.1005029.s003.docx]

# Supplementary Table 3: Percentage and breakdown of mortality by level of intellectual disability, disease categories and age group

|  | **Adults with severe or profound ID**  **(N=1,301) (%)** | **Adults with mild or moderate ID**  **(N=2,626) (%)** | **General adult population**  **(N=536,311) (%)** | ***p*-value**  **(severe/profound v mild/moderate)** | ***p*-value**  **(severe/profound v general population)** |
| --- | --- | --- | --- | --- | --- |
| **Aged 20-24** |  |  |  |  |  |
| All causes of death | 75 (100.0) | 36 (100.0) | 1246 (100.0) |  |  |
| Nervous system | 29 (38.7) | 11 (30.6) | 107 (8.6) | 0.405 | **<0.001** |
| Respiratory system | 7 (9.3) | 5 (13.9) | 29 (2.3) | 0.469 | **<0.01** |
| Congenital malformations, deformations, and chromosomal abnormalities | 11 (14.7) | # | 26 (2.1) | 0.347 | **<0.001** |
| Neoplasms | # | 5 (13.9) | 107 (8.6) | 0.059 | 0.163 |
| Circulatory system | # | 6 (16.7) | 72 (5.8) | **0.022** | 0.518 |
| **Aged 25-49** |  |  |  |  |  |
| All causes of death | 326 (100.0) | 332 (100.0) | 20,846 (100.0) |  |  |
| Nervous system | 118 (36.2) | 45 (13.6) | 688 (3.3) | **<0.001** | **<0.001** |
| Respiratory system | 30 (9.2) | 30 (9.0) | 705 (3.4) | 0.941 | **<0.001** |
| Congenital malformations, deformations, and chromosomal abnormalities | 40 (12.3) | 41 (12.3) | 193 (0.9) | 0.975 | **<0.001** |
| Neoplasms | 28 (8.6) | 55 (16.6) | 4,754 (22.8) | **<0.01** | **<0.001** |
| Circulatory system | 19 (5.8) | 56 (16.9) | 3,489 (16.7) | **<0.001** | **<0.001** |
| **Aged 50-64** |  |  |  |  |  |
| All causes of death | 506 (100.0) | 948 (100.0) | 57,484 (100.0) |  |  |
| Nervous system | 91 (18.0) | 82 (8.6) | 1,959 (3.4) | **<0.001** | **<0.001** |
| Respiratory system | 89 (17.6) | 112 (11.8) | 4,392 (7.6) | **<0.01** | **<0.001** |
| Congenital malformations, deformations, and chromosomal abnormalities | 108 (21.3) | 206 (21.7) | 430 (0.7) | 0.865 | **<0.001** |
| Neoplasms | 63 (12.5) | 165 (17.4) | 21,738 (37.8) | **0.013** | **<0.001** |
| Circulatory system | 54 (10.7) | 162 (17.1) | 13,946 (24.3) | **<0.01** | **<0.001** |
| **Aged 65+** |  |  |  |  |  |
| All causes of death | 394 (100.0) | 1,310 (100.0) | 456,735 (100.0) |  |  |
| Nervous system | 55 (14.0) | 112 (8.5) | 36,244 (7.9) | **<0.01** | **0.029** |
| Respiratory system | 90 (22.8) | 281 (21.5) | 54,233 (11.9) | 0.557 | **<0.001** |
| Congenital malformations, deformations, and chromosomal abnormalities | 34 (8.6) | 60 (4.6) | 404 (0.1) | **<0.01** | **<0.001** |
| Neoplasms | 45 (11.4) | 219 (16.7) | 116,230 (25.4) | **0.011** | **<0.001** |
| Circulatory system | 59 (15.0) | 259 (19.8) | 113,639 (24.9) | **0.032** | **<0.001** |
| **All adults (20+)** |  |  |  |  |  |
| All causes of death | 1,301 (100.0) | 2626 (100.0) | 536,311 (100.0) |  |  |
| Nervous system | 293 (22.5) | 250 (9.5) | 38,998 (7.3) | **<0.001** | **<0.001** |
| Respiratory system | 216 (16.6) | 428 (16.3) | 59,359 (11.1) | 0.809 | **<0.001** |
| Congenital malformations, deformations, and chromosomal abnormalities | 193 (14.8) | 309 (11.8) | 1,053 (0.2) | **<0.01** | **<0.001** |
| Neoplasms | 139 (10.7) | 444 (16.9) | 142,829 (26.6) | **<0.001** | **<0.001** |
| Circulatory system | 134 (10.3) | 483 (18.4) | 131,146 (24.5) | **<0.001** | **<0.001** |

Data are presented as numbers (percentages of deaths in each age group). ID = intellectual disability. #Counts <5 are suppressed. *p* values were derived using the chi-squared test.
