# Supplementary material for: Avoidable mortality, clinical outcomes and determinants of premature death among adults with severe or profound intellectual disability: A population-based analysis of mortality data in England
Source: PLoS Med. 2026 Aug 17;23(8):e1005029. doi: 10.1371/journal.pmed.1005029 (PMC13480647; doi:10.1371/journal.pmed.1005029)
Supplement: Supplementary file 4 [file pmed.1005029.s004.docx]

# Supplementary Table 4: Years of life lost in adults with severe or profound intellectual disability by disease category (ICD-10 chapters and OECD definition) and sex

|  | Year of life lost | | | |
| --- | --- | --- | --- | --- |
|  | Female | Male | Unknown | Total |
| **All causes of death** | **17,636.7 (100.0)** | **19,119.7 (100.0)** | **288.4 (100.0)** | **37,044.7 (100.0)** |
| Nervous system | 4,739.7 (26.9) | 5,368.1 (28.1) | 91.3 (31.7) | 10,199.1 (27.5) |
| Congenital malformations, deformations, and chromosomal abnormalities | 2,526.5 (14.3) | 3,147.0 (16.5) | 13.8 (4.8) | 5,687.2 (15.4) |
| Respiratory system | 2,214.3 (12.5) | 2,860.1 (15.0) | 21.5 (7.5) | 5,095.9 (13.8) |
| Neoplasms | 2,033.4 (11.5) | 1,506.8 (7.9) | 0.0 (0.0) | 3,540.2 (9.6) |
| Circulatory system | 1,211.7 (6.9) | 1,818.6 (9.5) | 41.6 (14.4) | 3,071.9 (8.3) |
| **All causes of avoidable death** | **7,111.4 (40.3)** | **7,845.2 (41.0)** | **102.8 (35.6)** | **15,059.4 (40.7)** |
| Epilepsy | 1,107.5 (6.3) | 1,325.9 (6.9) | 62.9 (21.8) | 2,496.3 (6.7) |
| Pneumonia, not elsewhere classified or organism unspecified | 927.8 (5.3) | 1,215.5 (6.4) | 0.0 (0.0) | 2,143.3 (5.8) |
| Cerebrovascular diseases | 372.8 (2.1) | 675.3 (3.5) | 0.0 (0.0) | 1,048.1 (2.8) |
| COVID-19 | 641.1 (3.6) | 322.2 (1.7) | 0.0 (0.0) | 963.3 (2.6) |
| Ischaemic heart diseases | 245.9 (1.4) | 438.6 (2.3) | 0.0 (0.0) | 684.5 (1.8) |
| Lung diseases due to external agents | 235.4 (1.3) | 428.4 (2.2) | 0.0 (0.0) | 663.8 (1.8) |

Data are presented in years of life lost (percentage of all causes of death by each sex). ICD-10 = International Classification of Diseases, 10th Revision; OECD = Organisation for Economic Co-operation and Development.
