# Supplementary material for: Avoidable mortality, clinical outcomes and determinants of premature death among adults with severe or profound intellectual disability: A population-based analysis of mortality data in England
Source: PLoS Med. 2026 Aug 17;23(8):e1005029. doi: 10.1371/journal.pmed.1005029 (PMC13480647; doi:10.1371/journal.pmed.1005029)
Supplement: Supplementary file 5 [file pmed.1005029.s005.docx]

Supplementary Table 5: Cox regression of factors associated with age at death among adults with intellectual disability

| **Factors** | **Unadjusted HR (95% CI)** | ***p*-value** | **Level of ID adjusted HR**  **(95% CI)** | ***p*-value** | **Ethnicity adjusted HR (95% CI)** | ***p*-value** | **Level of ID and ethnicity adjusted HR (95% CI)** | ***p*-value** |
| --- | --- | --- | --- | --- | --- | --- | --- | --- |
| **All adults with ID (N=3,927)** | | | | | | | | |
| **Level of ID** | | | | | | | | |
| Mild or moderate (reference) | - | - | - | - | - | - | - | - |
| Severe or profound | **1.52**  **(1.42, 1.63)** | **<0.001** | - | - | **1.47**  **(1.37, 1.57)** | **<0.001** | - | - |
| **Ethnicity** | | | | | | | | |
| White (reference) | - | - | - | - | - | - | - | - |
| Ethnic groups other than  White ethnicity | **2.62**  **(2.31, 2.97)** | **<0.001** | **2.49**  **(2.19, 2.82)** | **<0.001** | - | - | - | - |
| **Sex** | | | | | | | | |
| Female (reference) | - | - | - | - | - | - | - | - |
| Male | 1.03  (0.96, 1.09) | 0.439 | 1.04  (0.98, 1.11) | 0.228 | 1.00  (0.94, 1.07) | 0.97 | 1.02  (0.95, 1.09) | 0.600 |
| **Index of multiple deprivation (IMD)** | | | | | |  |  |  |
| 9-10 (least deprived, reference) | - | - | - | - | - | - | - | - |
| 7-8 | 1.01  (0.90, 1.14) | 0.830 | 1.00  (0.89, 1.13) | 0.951 | 1.02  (0.91, 1.15) | 0.701 | 1.01  (0.90, 1.14) | 0.873 |
| 5-6 | 1.06  (0.95, 1.18) | 0.328 | 1.09  (0.97, 1.22) | 0.143 | 1.06  (0.95, 1.19) | 0.286 | 1.09  (0.97, 1.22) | 0.137 |
| 3-4 | 1.01  (0.90, 1.12) | 0.935 | 1.03  (0.92, 1.15) | 0.662 | 0.98  (0.87, 1.09) | 0.696 | 0.99  (0.89, 1.11) | 0.866 |
| 1-2 (most deprived) | 1.09  (0.98, 1.22) | 0.108 | **1.12**  **(1.00, 1.24)** | **0.047** | 1.09  (0.98, 1.21) | 0.134 | 1.10  (0.99, 1.23) | 0.081 |
| **Adults with severe or profound ID (N=1,301)** | | | | | | | | |
| **Ethnicity** | | | | | | | | |
| White (reference) | - | - | - | - | - | - |  | - |
| Ethnic groups other than  White ethnicity | **2.48**  **(2.08, 2.96)** | **<0.001** | - | - | - | - | - | - |
| **Sex** | - | - | - | - | - | - | - | - |
| Female (reference) | - | - | - | - | - | - | - | - |
| Male | 1.03  (0.93, 1.15) | 0.546 | - | - | 1.02  (0.91, 1.14) | 0.707 | - | - |
| **Index of multiple deprivation (IMD)** | | | | | | | | |
| 9-10 (least deprived, reference) | - | - | - | - | - | - | - | - |
| 7-8 | 0.94  (0.78, 1.14) | 0.541 | - | - | 0.91  (0.75, 1.11) | 0.346 | - | - |
| 5-6 | 1.15  (0.96, 1.39) | 0.132 | - | - | 1.14  (0.94, 1.37) | 0.190 | - | - |
| 3-4 | 0.98  (0.82, 1.19) | 0.858 | - | - | 0.90  (0.75, 1.09) | 0.288 | - | - |
| 1-2 (most deprived) | 1.05  (0.88, 1.26) | 0.596 | - | - | 1.00  (0.83, 1.20) | 0.956 | - | - |

CI = confidence interval, HR = hazard ratio, ID = intellectual disability, IMD = index of multiple deprivation. *p* values were derived using the Cox regression.
