# Supplementary material for: Avoidable mortality, clinical outcomes and determinants of premature death among adults with severe or profound intellectual disability: A population-based analysis of mortality data in England
Source: PLoS Med. 2026 Aug 17;23(8):e1005029. doi: 10.1371/journal.pmed.1005029 (PMC13480647; doi:10.1371/journal.pmed.1005029)
Supplement: Supplementary file 6 [file pmed.1005029.s006.docx]

Supplementary Table 6: Cox regression of factors associated with age at death among adults with intellectual disability and delayed entry (left truncation) on 1 January 2021

| **Factors** | **Unadjusted HR (95% CI)** | ***p*-value** | **Level of ID adjusted HR**  **(95% CI)** | ***p*-value** | **Ethnicity adjusted HR (95% CI)** | ***p*-value** | **Level of ID and ethnicity adjusted HR (95% CI)** | ***p*-value** |
| --- | --- | --- | --- | --- | --- | --- | --- | --- |
| **All adults with ID (N=3,927)** | | | | | | | | |
| **Level of ID** | | | | | | | | |
| Mild or moderate (reference) | - | - | - | - | - | - | - | - |
| Severe or profound | 1.04  (0.97, 1.11) | 0.271 | - | - | 1.04  (0.97, 1.11) | 0.316 | - | - |
| **Ethnicity** | | | | | | | | |
| White (reference) | - | - | - | - | - | - | - | - |
| Ethnic groups other than  White ethnicity | 1.10  (0.96, 1.25) | 0.167 | 1.09  (0.96, 1.24) | 0.186 | - | - | - | - |
| **Sex** | | | | | | | | |
| Female (reference) | - | - | - | - | - | - | - | - |
| Male | 1.01  (0.95, 1.07) | 0.796 | 1.01  (0.95, 1.08) | 0.773 | 1.01  (0.94, 1.07) | 0.835 | 1.01  (0.94, 1.07) | 0.814 |
| **Index of Multiple Deprivation (IMD)** | | | | | |  |  |  |
| 9-10 (least deprived, reference) | - | - | - | - | - | - | - | - |
| 7-8 | 1.03  (0.92, 1.16) | 0.576 | 1.03  (0.92, 1.16) | 0.572 | 1.03  (0.91, 1.16) | 0.626 | 1.03  (0.91, 1.16) | 0.623 |
| 5-6 | 1.02  (0.91, 1.14) | 0.688 | 1.03  (0.92, 1.15) | 0.662 | 1.02  (0.91, 1.14) | 0.715 | 1.02  (0.91, 1.15) | 0.689 |
| 3-4 | 1.03  (0.92, 1.15) | 0.637 | 1.03  (0.92, 1.15) | 0.602 | 1.02  (0.91, 1.14) | 0.698 | 1.03  (0.92, 1.15) | 0.663 |
| 1-2 (most deprived) | 1.03  (0.93, 1.15) | 0.565 | 1.03  (0.93, 1.15) | 0.532 | 1.03  (0.92, 1.15) | 0.597 | 1.03  (0.93, 1.15) | 0.565 |
| **Adults with severe or profound ID (N=1,301)** | | | | | | | | |
| **Ethnicity** | | | | | | | | |
| White (reference) | - | - | - | - | - | - |  | - |
| Ethnic groups other than  White ethnicity | 1.07  (0.89, 1.29) | 0.463 | - | - | - | - | - | - |
| **Sex** | - | - | - | - | - | - | - | - |
| Female (reference) | - | - | - | - | - | - | - | - |
| Male | 0.99  (0.88, 1.10) | 0.803 | - | - | 0.98  (0.88, 1.10) | 0.737 | - | - |
| **Index of Multiple Deprivation (IMD)** | | | | | | | | |
| 9-10 (least deprived, reference) | - | - | - | - | - | - | - | - |
| 7-8 | 1.04  (0.86, 1.26) | 0.679 | - | - | 1.03  (0.85, 1.26) | 0.750 | - | - |
| 5-6 | 1.05  (0.87, 1.27) | 0.606 | - | - | 1.05  (0.87, 1.27) | 0.600 | - | - |
| 3-4 | 1.07  (0.88, 1.30) | 0.483 | - | - | 1.07  (0.88, 1.30) | 0.497 | - | - |
| 1-2 (most deprived) | 1.05  (0.88, 1.27) | 0.571 | - | - | 1.05  (0.88, 1.27) | 0.576 | - | - |

CI = confidence interval, HR = hazard ratio, ID = intellectual disability, IMD = index of multiple deprivation. *p* values were derived using the Cox regression.
